# Supplementary material for: Different laterality indexes are poorly correlated with one another but consistently show the tendency of males and females to be more left- and right-lateralized, respectively
Source: R Soc Open Sci. 2020 Apr 15;7(4):191700. doi: 10.1098/rsos.191700 (PMC7211879; doi:10.1098/rsos.191700)
Supplement: Supplementary Material, Figures and Tables [file rsos191700supp1.docx]

**SUPPLEMENTARY MATERIALS**

**Different laterality indexes are poorly correlated with one another but consistently show the tendency of males and females to be more left- and right- lateralised, respectively**

Carlos Buenaventura Castillo^1,2^, Andy Graeme Lynch^1,2^, Silvia Paracchini^1^*

*correspondence to: [sp58@st-andrews.ac.uk](mailto:sp58@st-andrews.ac.uk)

**Supplementary Methods**

Extracts from the ALSPAC documentation available here (<http://www.bristol.ac.uk/alspac/researchers/our-data/>) describing the tasks used in this study.

**Grip strength**

The child sat in a chair with arms and back support and was asked to rest his/her forearms on the arms of the chair with their wrist just over the end of the arm of the chair. The wrist placed in a neutral position with the thumb facing upwards.

The tester demonstrated how to use the dynamometer to the child showing how gripping very tightly registered the best score. They explained to the child, that squeezing the dynamometer feels slightly strange as nothing actually moves. (The dynamometer registers the grip on the dial but the actual metal component of the machine doesn’t move).

The child was given a practice squeeze of the dynamometer to ensure that it felt comfortable. Starting with the right hand, the hand was positioned so that the thumb was round one side of the handle and the four fingers were around the other side. It was important that the instrument felt comfortable for the child and the position of the handle was altered if necessary. The measurer rested the base of the dynamometer on the palm of the child’s hand in order to support the weight of the dynamometer, whilst ensuring that the movement of the machine was not restricted. The child was encourage to squeeze as long and as tightly as possible or until the needle stopped rising. The higher the reading, the stronger the grip.

The child repeated the measurement with the left hand and two further measurements were taken with each hand, alternating sides to give three readings in total for each side. Derived variables have been computed indicating the mean value for the right and left hand.

**Manual dexterity: Placing Pegs**

The Movement Assessment Battery for Children (Movement ABC, Henderson & Sugden, 1992) was used to test the children’s motor ability. It comprises three sections, assessing static and dynamic balance, manual dexterity and ball skills. Because of time constraints, it was not possible to conduct the whole assessment, so specific subtests from each of the three sections were carried out.

In the placing pegs task (known in the clinic as the peg game), the child had to insert twelve pegs, one at a time, into a peg board, holding the board with one hand and inserting the pegs with the other, as quickly as possible. The task was carried out with the preferred and the non-

preferred hand, after it had been described and demonstrated by the tester, and after a practice attempt with each hand. Time taken to complete the task was recorded for each hand. If the child failed to carry out the instructions, for example, picked up more than one peg at a time, or used both hands to place the pegs, he or she failed that trial and a second trial with the same hand was given. Variables have been created which summarise the child’s performance over the different trials for the preferred and non-preferred hand.

**Laterality: mark squares and sorting matches**

Two tests of laterality were undertaken; one using ticks to mark squares on a piece of paper the other moving matches. Both tasks are a repetition of those used in the National Child Development Study, 1958 Cohort (see Leask and Crow, 2001). The Child had the tasks explained to them and were told that they would have to do each task twice, once using each hands. The child was asked to indicate which was their preferred hand before the tasks began.

Mark squares: The child is asked to make a short dash on a piece of paper which has a grid marked on it consisting of rows of 20 squares. They are asked to start at the top left hand side of the squared paper, working across it. When the first line is completed the child should start on the left side of the next row. A pencil was used and the object was to see how many squares can be marked in 60 seconds. The task was first demonstrated by the tester on a small grid the child had a practice. When the child has completed the test with the preferred hand, they change hands and turn the paper around and repeat. The tester counts the number of squares completed with each hand.

Sorting matches: The child is asked to sit at a table on which is a board that has two match boxes 12 inches apart fixed on to it. The child is asked to take the matches one at a time out of the full box (on their right,) and transfer them directly to the empty box (on their left) using one hand only, starting with the preferred hand. (The other hand may be used to steady either box). The children were allowed one practice go before they started the task. The tester timed how long it took for the child to transfer all the matches from one box to the other. The test was then repeated with the non-preferred hand.

**Supplementary Table S1. Package versions**

| **Package** | **Version** | **Built** |
| --- | --- | --- |
| car | 3.0-6 | 3.6.2 |
| foreign | 0.8-74 | 3.6.2 |
| GGally | 1.4.0 | 3.6.1 |
| ggfortify | 0.4.8 | 3.6.2 |
| ggpubr | 0.2.4 | 3.6.2 |
| gplots | 3.0.1.2 | 3.6.1 |
| gridExtra | 2.3 | 3.6.1 |
| ltm | 1.1-1 | 3.6.1 |
| moments | 0.14 | 3.6.0 |
| Publish | 2019.12.04 | 3.6.2 |
| reshape | 0.8.8 | 3.6.1 |
| rlang | 0.4.2 | 3.6.2 |
| tidyverse | 1.3.0 | 3.6.2 |
| visdat | 0.5.3 | 3.6.1 |
| xlsx | 0.6.1 | 3.6.0 |
| xlsx | 0.6.1 | 3.6.0 |
| foreign | 0.8-72 | 3.6.2 |

**Supplementary Table S2. Comparison of age in males and females**

|  | **Males** | | | **Females** | | | **Welch** | ${\bar{\boldsymbol{x}}}_{\boldsymbol{m}}\boldsymbol{-}{\bar{\boldsymbol{x}}}_{\boldsymbol{f}}$ |
| --- | --- | --- | --- | --- | --- | --- | --- | --- |
|  | $\boldsymbol{n}_{\boldsymbol{m}}$ | ${\bar{\boldsymbol{x}}}_{\boldsymbol{m}}$ | $\boldsymbol{se}_{\boldsymbol{m}}$ | $\boldsymbol{n}_{\boldsymbol{f}}$ | ${\bar{\boldsymbol{x}}}_{\boldsymbol{f}}$ | $\boldsymbol{se}_{\boldsymbol{f}}$ | **t-test**  ***P-val*** | **95% c.i.** |
| **PegQ** | 3,465 | 2,768.12 | 117.73 | 3,419 | 2,769.95 | 118.2 | 0.5196 | (-7.41, 3.74) |
| **MarkQ** | 3,630 | 3,901.53 | 95.06 | 3,759 | 3,901.83 | 96.34 | 0.8937 | (-4.66, 4.07) |
| **SortQ** | 3,627 | 3,901.54 | 95.33 | 3,739 | 3,901.69 | 96.35 | 0.9475 | (-4.53, 4.23) |
| **GripQ** | 3,271 | 4,302.75 | 86.16 | 3,393 | 4,304.11 | 88.19 | 0.5235 | (-5.55, 2.82) |

*Age is measured in days*


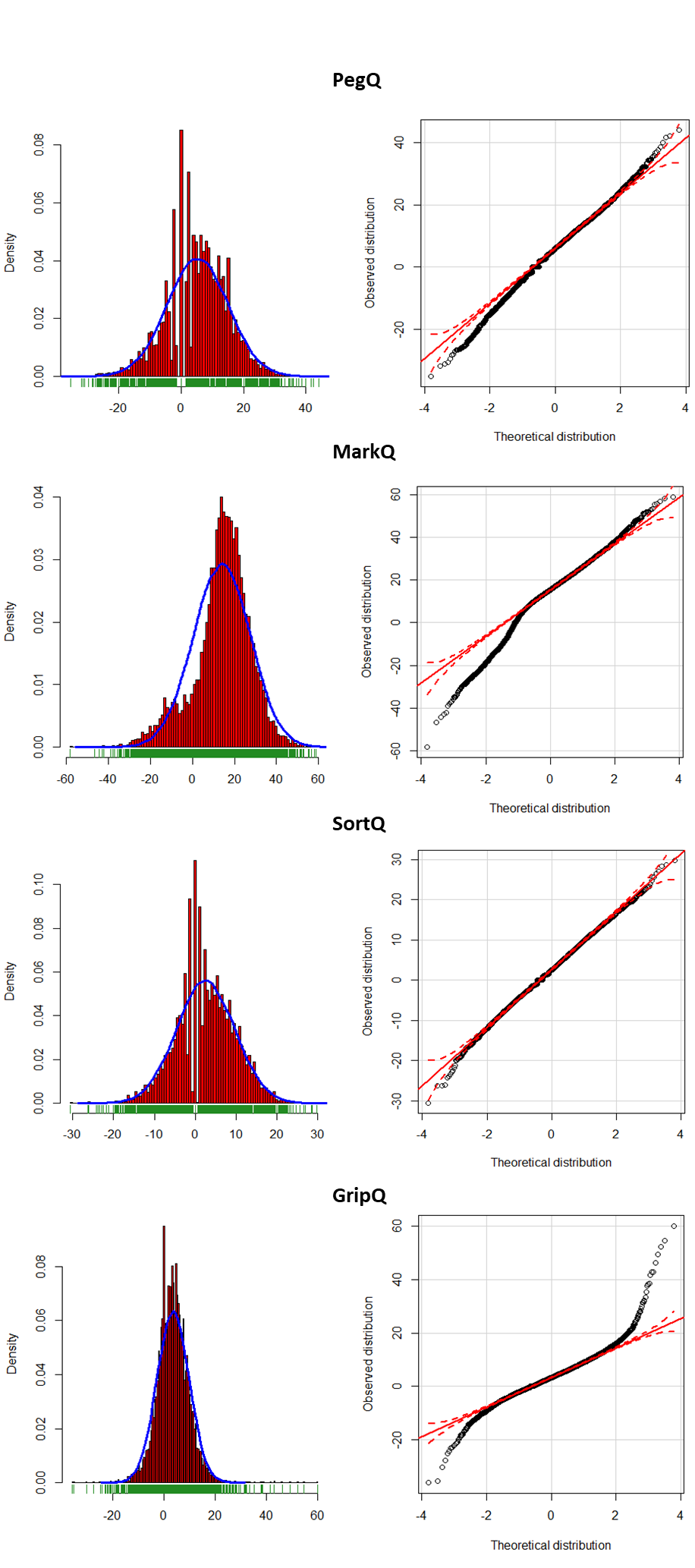


**Supplementary Figure S1** Laterality indexes. Histograms and Q-Q plots for PegQ, MarkQ, SortQ and GripQ. Each individual measure is represented by the green rugs at the bottom of each distribution. The blue lines describe normal distributions under the observed mean and variance. The Q-Q plots illustrate the differences between a normal (red lines) and the observed (black circles) distribution. The PegQ and SortQ distributions showed lack of observations around zero for all indexes, but not at zero. A score of zero was achieved when both hands scored the same time. The next smallest difference was measured in seconds, without decimals. Therefore, a score close to zero [1/(L+R)] could only be achieved with a high value at the denominator given by the sum of the score for the two hands, for which however there is a ceiling effect.


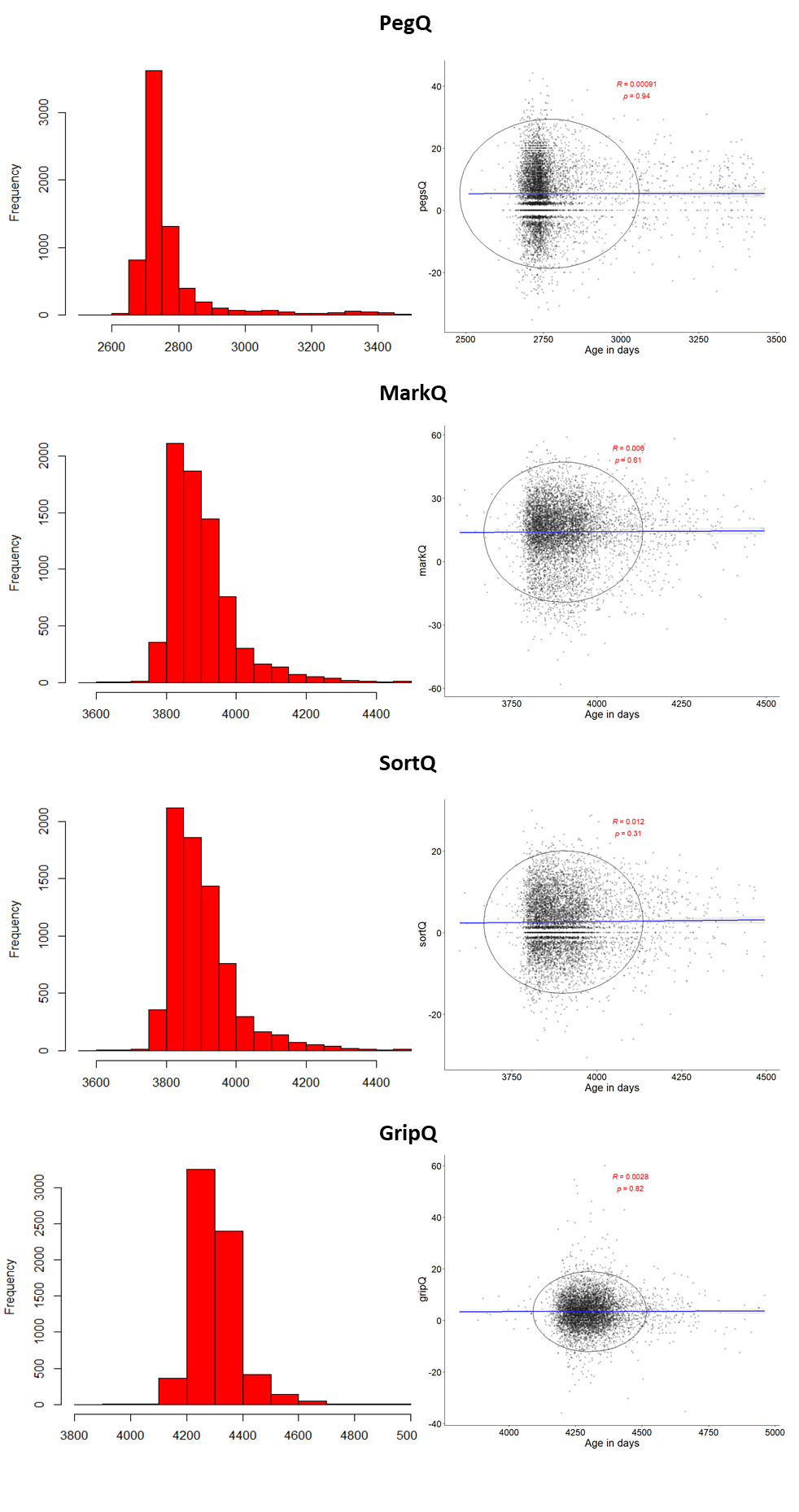


**Supplementary Figure S2. Age effect on laterality indexes.** The histograms on the left show the age, measured in days, of participants when data were collected. On the right, the plots the laterality indexes are plotted against the age measured days. The regression lines (in blue) show no age effects on the indexes (statistical values reported in red).

**A)**


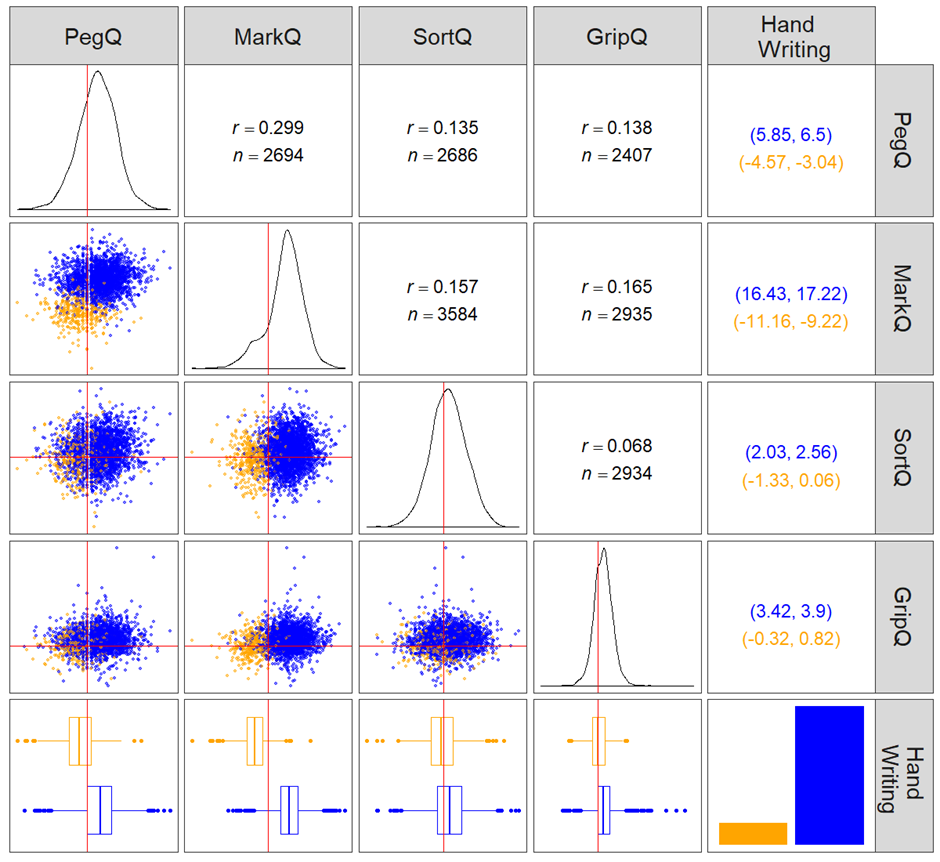


**B)**


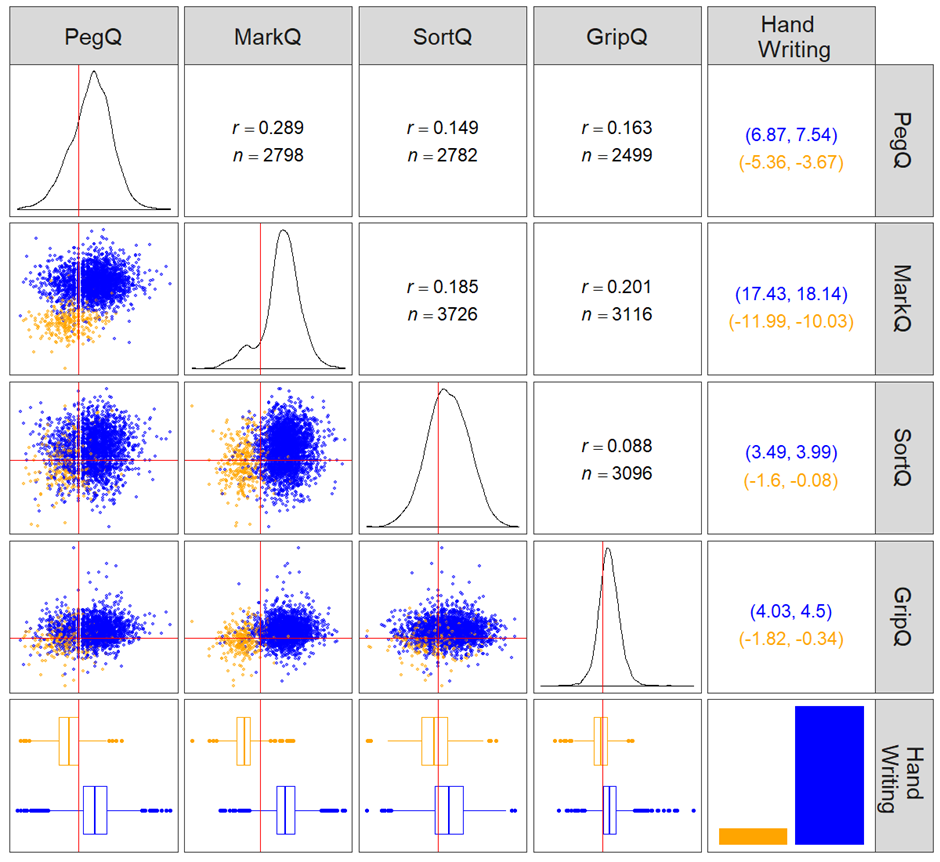


**Supplementary Figure S3** Correlation of laterality measures in A) males and B) females. The cells along the diagonal show the empirical distribution for each index and the bar-plot for the preferred hand for writing. The boxes on the left of the main diagonal show the bivariate distribution of the indexes colour coded for preferred hand for writing (left = orange; right =blue). For example, the first box on the second raw of the matrix illustrates the PegQ scores on the x-axis and MarkQ on the y-axis. The bottom row shows the box-plots for each laterality index grouped by preferred hand for writing. The red lines are aligned along the zero for each index. The cells on the right of the diagonal show the correlation coefficients and the sample sizes from which they were calculated. The last column reports the confidence intervals for the box-plots shown in the bottom row, providing a measure on how the different indexes separate the right and left handers for preferred writing hand.

**A)**


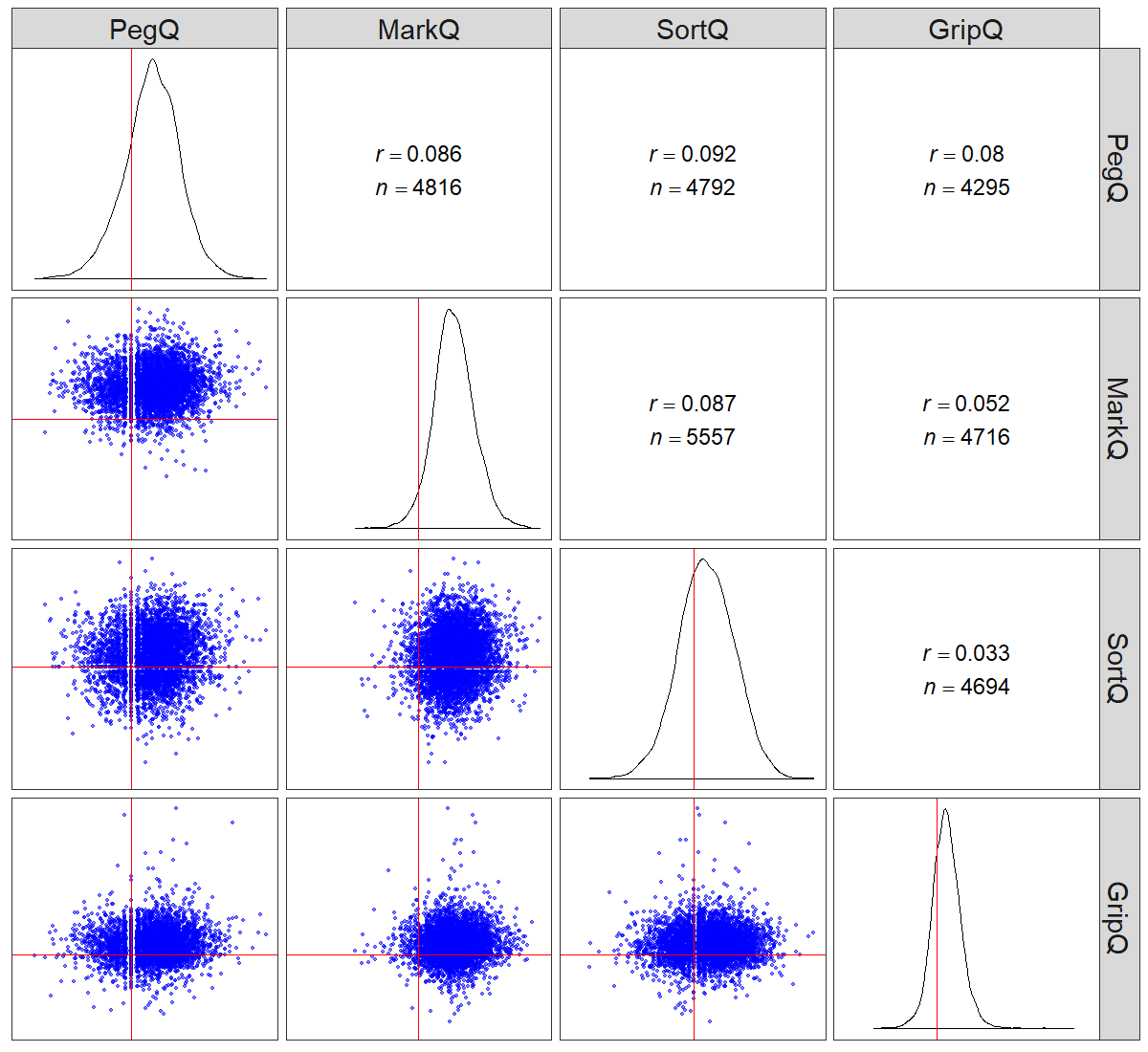


**B)**


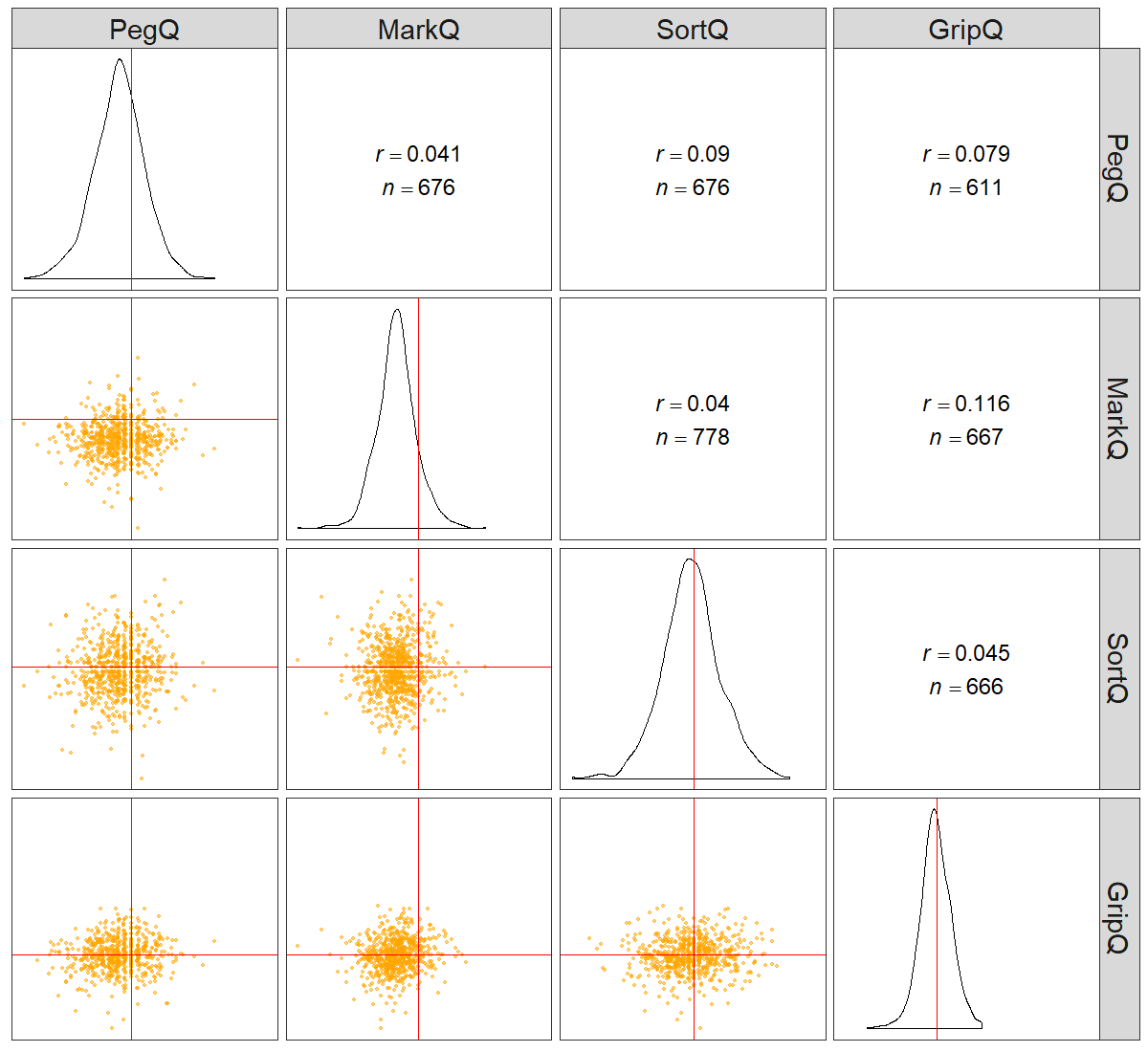


**Supplementary Figure S4** Correlation of laterality measures in A) right and B) left handers defined by preferred hand for writing. The cells along the diagonal show the empirical distribution for each index and the bar-plot for the preferred hand for writing. The boxes on the left of the main diagonal show the distribution of the indexes. The red lines are aligned along the zero for each index. The cells on the right of the diagonal show the correlation coefficients and the sample sizes from which they were calculated.


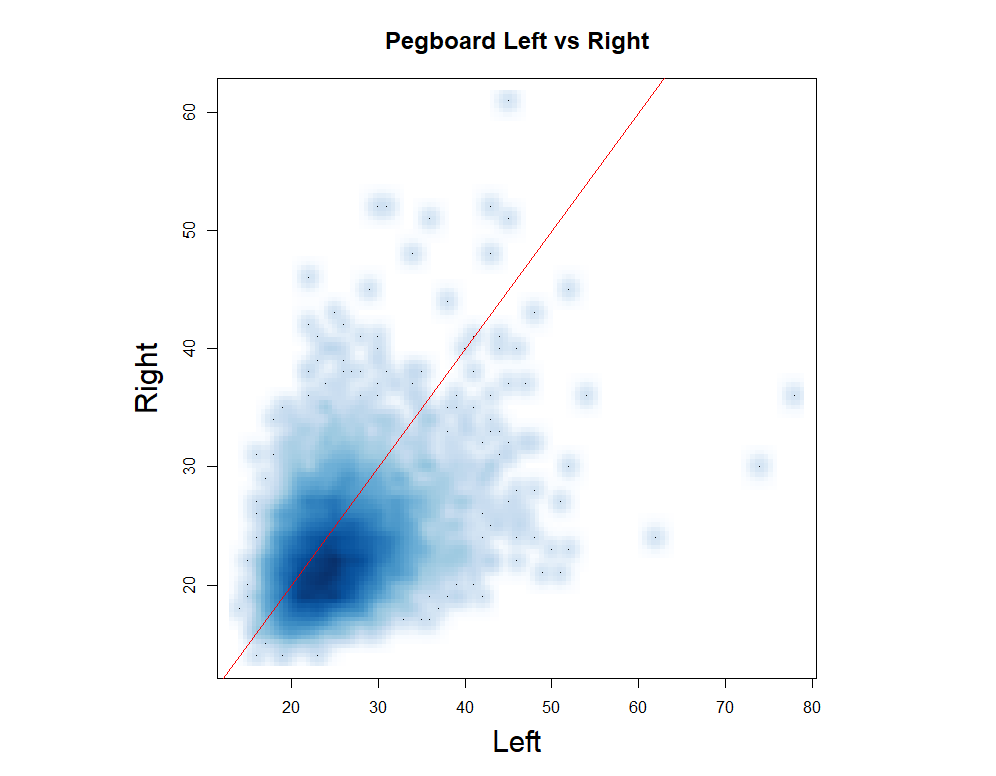

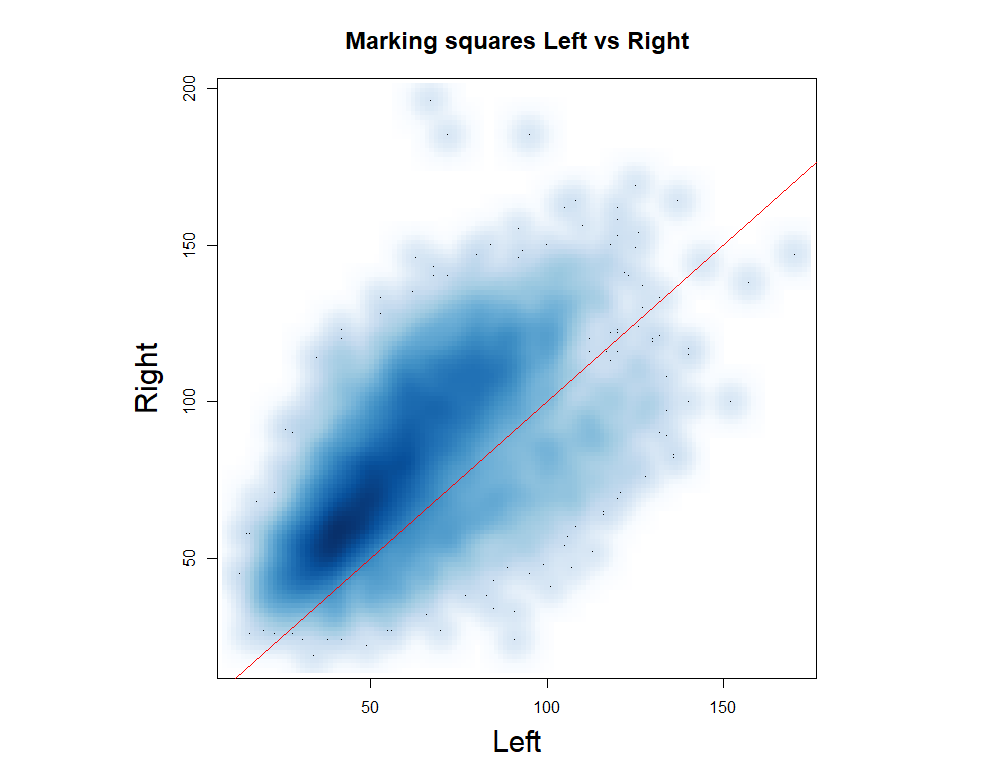

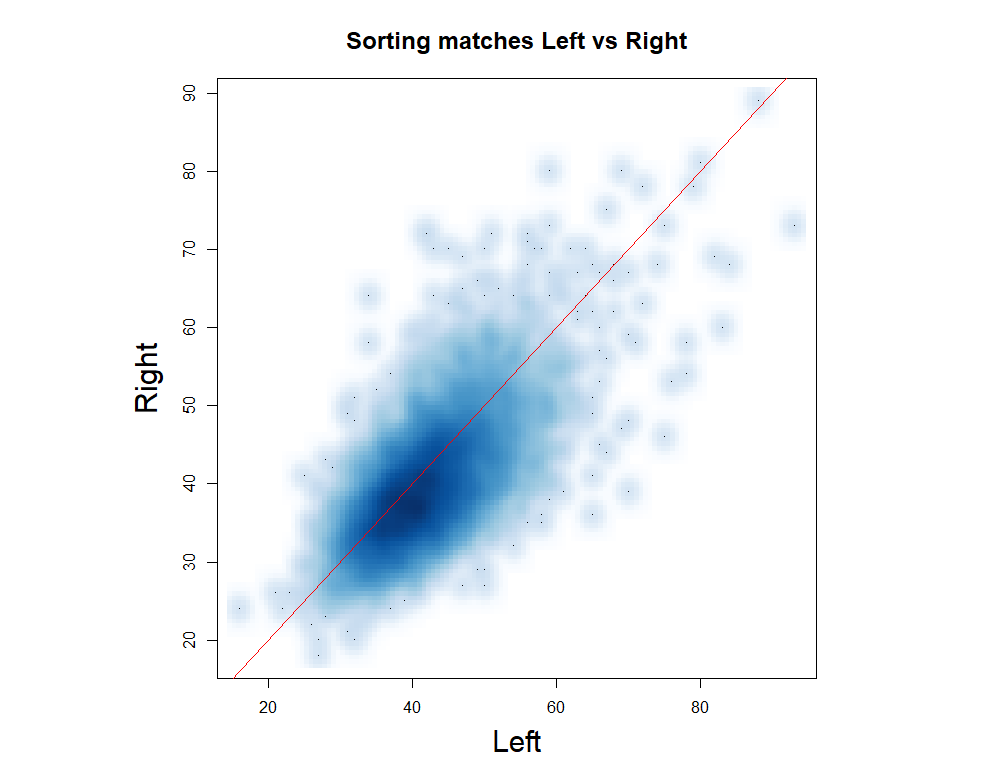

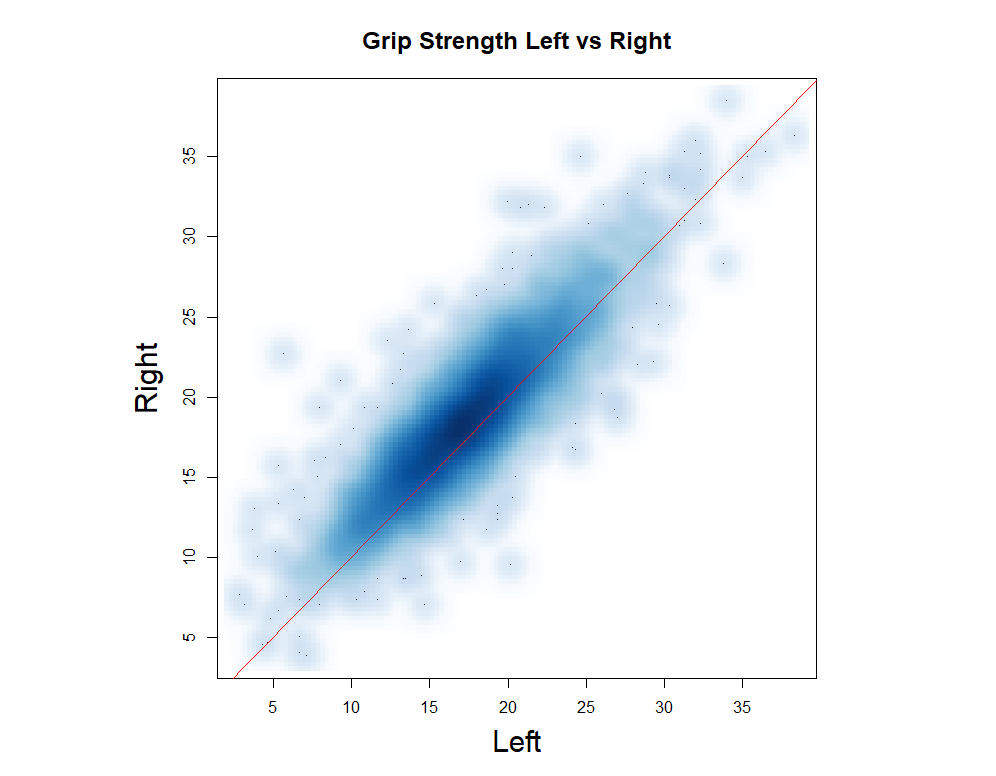


**Supplementary Figure S5 Performance of both hands.** The performance of both the right and left hand is plotted for each individuals in the four tasks: Pegboarg, Marking Squares, Sorting Matches and Grip strength. Equal performance of both hands would plot along the red line.

**
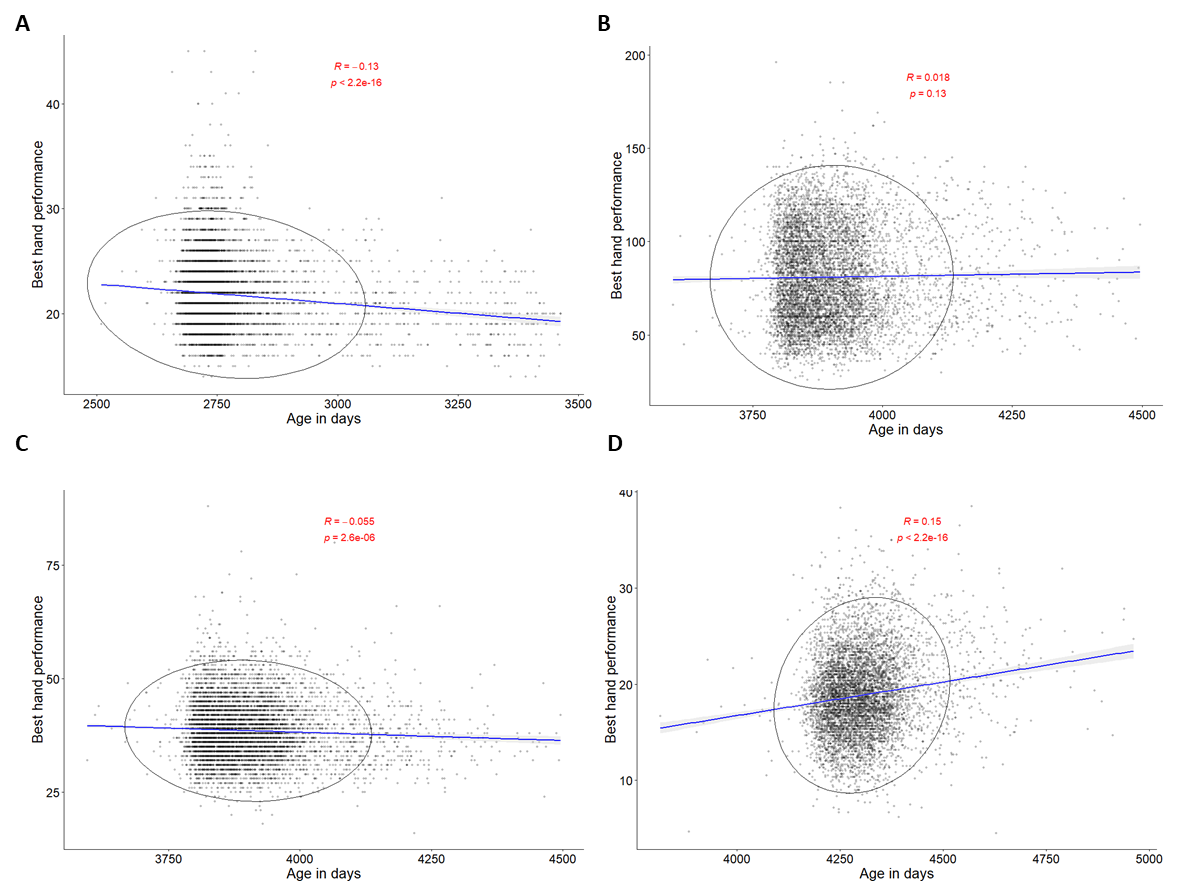
**

**Supplementary Figure S6 Age effect on best performance.** The best performance regardless of left or right preference was plotted against age for A) Pegboard, B) Marking squares, C) Sorting matches and D) Grip strength. Correlation and statistical significance are shown in red and a regression line is shown in blue.
